# Minding the gap and overlap: a literature review of fragmentation of primary care for chronic dialysis patients

**DOI:** 10.1186/s12882-017-0689-0

**Published:** 2017-08-29

**Authors:** Virginia Wang, Clarissa J. Diamantidis, JaNell Wylie, Raquel C. Greer

**Affiliations:** 1 Division of General Internal Medicine, Department of Medicine, Duke University School of Medicine, Durham, 27710 NC USA; 2 Department of Population Health Sciences, Duke University School of Medicine, Durham, NC USA; 3 Center for Health Services Research in Primary Care, Durham Veterans Affairs Health Care System, Durham, NC USA; 4 Division of Nephrology, Department of Medicine, Duke University School of Medicine, Durham, NC USA; 5 Department of Orthopaedics, Duke University School of Medicine, Durham, NC USA; 6 Division of General Internal Medicine, Department of Medicine, Johns Hopkins University School of Medicine, Baltimore, MD USA

**Keywords:** Primary care services, Maintenance dialysis, Physicians, Coordination of care, Outcomes

## Abstract

**Background:**

Care coordination is a challenge for patients with kidney disease, who often see multiple providers to manage their associated complex chronic conditions. Much of the focus has been on primary care physician (PCP) and nephrologist collaboration in the early stages of chronic kidney disease, but less is known about the co-management of the patients in the end-stage of renal disease. We conducted a systematic review and synthesis of empirical studies on primary care services for dialysis patients.

**Methods:**

Systematic literature search of MEDLINE/PubMED, CINAHL, and EmBase databases for studies, published until August 2015. Inclusion criteria included publications in English, empirical studies involving human subjects (e.g., patients, physicians), conducted in US and Canadian study settings that evaluated primary care services in the dialysis patient population.

**Results:**

Fourteen articles examined three major themes of primary care services for dialysis patients: perceived roles of providers, estimated time in providing primary care, and the extent of dialysis patients’ use of primary care services. There was general agreement among providers that PCPs should be involved but time, appropriate roles, and miscommunication are potential barriers to good primary care for dialysis patients. Although many dialysis patients report having a PCP, the majority rely on primary care from their nephrologists. Studies using administrative data found lower rates of preventive care services than found in studies relying on provider or patient self-report.

**Discussion:**

The extant literature revealed gaps and opportunities to optimize primary care services for dialysis patients, foreshadowing the challenges and promise of Accountable Care / End-Stage Seamless Care Organizations and care coordination programs currently underway in the United States to improve clinical and logistical complexities of care for this commonly overlooked population. Studies linking the relationship between providers and patients’ receipt of primary care to outcomes will serve as important comparisons to the nascent care models for ESRD patients, whose value is yet to be determined.

## Background

Overall and disease-specific management and care coordination is a challenge for patients with complex and multiple chronic conditions (MCC), who typically see multiple providers to manage one or more co-morbid conditions. This problem is amplified for patients with kidney disease, whose condition is associated and co-morbid with highly prevalent conditions such as hypertension, diabetes, and heart disease. Much of the literature and intervention efforts have focused on primary care and nephrology physician collaborative care in the early stages of chronic kidney disease (CKD), with an emphasis on primary care physician (PCP) intervention to prevent, detect, treat, and slow the progression of kidney disease. Studies have generally found lack of clarity on the roles and responsibilities of clinical management among providers [1], poor communication and coordination between primary care physicians and nephrologists [2] and suboptimal general and CKD-related care in patients [3, 4].

However, less is known about primary care physician (PCP) and nephrologist co-management of patients in the end-stage of renal disease. Specifically, most patients with advanced kidney failure undergo chronic dialysis treatments and are clinically managed by clinical staff at dialysis facilities on a frequent basis (e.g., thrice weekly for in-center hemodialysis or monthly for home-based dialysis). Due in part to the rigidity of the dialysis treatment schedule, management of co-morbid illness and their complications by providers outside of the dialysis unit is challenging. Patients commonly receive supervision of non-renal health needs during their dialysis treatment visits due to convenience and familiarity. As a result, primary care management may default to renal providers. Compared to early-stage CKD care, this observed focus – of nephrologist involvement in treating primary care needs of patients with end-stage renal disease (ESRD) – raises important questions about the role of the PCP, traditionally considered the patient’s medical home for continuous comprehensive care and the “quarterback” responsible for assessing, balancing, and coordinating the care of patients’ multiple competing conditions [5, 6]. Who is responsible for and actively managing the primary care needs of these dialysis patients? Discrepant expectations and subsequent provision of primary care may exacerbate care fragmentation in this highly complex and vulnerable patient population, increasing the potential for unnecessary duplication of care or adverse outcomes. To date, PCP and nephrology roles in the actual provision of primary care services for chronic dialysis patients is not well-defined. We conducted a systematic review of primary care service provision for dialysis patients, in order to assess 1) patient and provider perceptions of PCPs and nephrologists roles; 2) the extent to which PCPs and nephrologists deliver primary care services to chronic dialysis patients; 3) reported barriers to patients’ receipt or physicians’ delivery of primary care services; and 4) the measures used to assess provider provision and primary care outcomes in dialysis patients.

Understanding the provision of primary care among nephrologists and PCPs, fragmentation of care, and outcomes in dialysis patients is an emerging and important concern [7]. The anticipated growth and surveillance of patients with CKD [8] and the diminishing supply of practicing nephrologists [9, 10] suggests that nephrologists may be unable to continue or increase their provision of primary care services to dialysis patients, and it remains to be seen if nephrologists’ provision of primary care services is adequate or appropriate. In addition, recent efforts to organize chronic disease management, such as multidisciplinary care teams and the patient-centered medical home, commonly exclude patients with ESRD who may also benefit from models designed to mitigate care fragmentation and improve coordination. Elucidating the types of services and providers managing dialysis patients’ primary care needs and their effectiveness will illuminate the gaps in our understanding of primary care and nephrologist collaboration to guide further research and intervention to improve care for this overlooked, high healthcare utilizing, co-morbid dialysis patient population.

## Methods

This literature review is guided by conceptual frameworks for care provided to individuals with MCC [11] and interprofessional and co-managed care [12]. Together, these frameworks illustrate the complexity of care for patients receiving maintenance dialysis and is a valuable lens to frame our understanding of how their specialized and general healthcare needs are met. Briefly, the National Quality Forum’s model for MCC care recognizes the various ways in which patient preferences, care settings and providers, and types of healthcare services interact to impact health outcomes (e.g., care coordination, prevention of disease, cost). The interaction between these domains of care is dynamic, as patients’ healthcare needs evolve over time [11]. Our review of the empirical literature focuses on the domains regarding dialysis patients’ providers, their use, and measures used to assess their primary care. We also incorporate perspectives from Retchin’s collaborative model of interprofessional and co-managed care, which posits that interaction and coordination between MCC provider types is influenced by temporality (e.g., concurrent vs. sequential care), urgency, and delineation of authority in patient care [12]. To this end, our review also considers the evidence on perceptions regarding provider roles and skills in primary care delivery.

### Definitions and eligibility criteria for relevant literature

In this review, variables of interest pertain to the provision of primary care services among patients undergoing chronic dialysis treatment. For the purpose of this structured literature review, we conducted a broad search of the two main terms of ‘dialysis’ and’ primary care’, due to the anticipated paucity of published studies in this area. We defined *dialysis patients* as those with ESRD and undergoing chronic dialysis treatment (inclusive of all modalities, in-center or home-based dialysis). *Primary care* is more loosely defined. We adopted the Institute of Medicine’s definition of *primary care’s scope of services* [13], which includes general health maintenance; prevention (e.g., immunizations) and early detection (e.g., screening for cancer, depression); counseling of patients (e.g., diet, nutrition, tobacco cessation); risk assessment; management of acute care and chronic care (e.g., diabetes, hypertension, heart disease), care coordination and referrals. As follows, *providers of primary care* services include physicians represented in internal medicine, family medicine, geriatric medicine, general medicine, and nephrology, as well as non-physician providers such as physician assistant, nurse practitioner, and registered nurse. Last, we considered several settings *where provision of primary care services take place*, including physician offices and clinics of internal, family, and geriatric medicine, nephrology clinics, and dialysis centers.

From the published literature, we applied several eligibility criteria for inclusion in critical synthesis of the literature. Publications in English, empirical studies involving human subjects (e.g., patients, physicians), conducted in US and Canadian study settings and published until August 2015 were included for review. Editorials, letters, and literature review articles, and duplicate citations and publications that did not evaluate primary care services in the dialysis patient population were excluded.

### Literature search and selection criteria

**Fig. 1:**
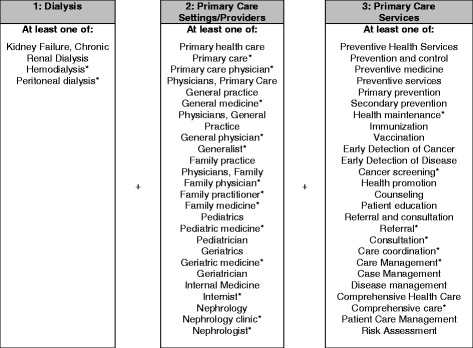
MeSH terms and keywords used in literature search. Notes: MeSH terms were also searched as keywords. *Denotes keyword

Literature included for synthesis were identified using 3 search strategies. The primary approach involved searching MEDLINE/PubMED, CINAHL, and Embase electronic databases for articles related to primary care services for dialysis patients. This search strategy employed numerous Medical Subject Headings (MeSH) and keywords related to primary care and dialysis patients (Fig. 1). Eligibility criteria were first applied to titles and abstracts and then full text review of potential references. To identify qualifying articles and ensure saturation of search results, the reference lists of eligible articles and literature reviews (that were not included in the literature synthesis) were examined. We applied similar eligibility criteria to these references through this backward search method. Inclusion and exclusion criteria were applied by four reviewers and dual coding of all citations found 98.7% agreement in title and abstract reviews and 90.2% in full text reviews. Disagreements were resolved by discussion and consensus among all four reviewers.

### Data collection and analysis

Key information in each article was extracted by 2 reviewers (VW, RCG). Data included overall study design, study setting and participants, and key measures. Key variables of interest included the types of providers, primary care services, and outcomes that were assessed or reported and authors’ results.

## Results

### Literature search

**Fig. 2:**
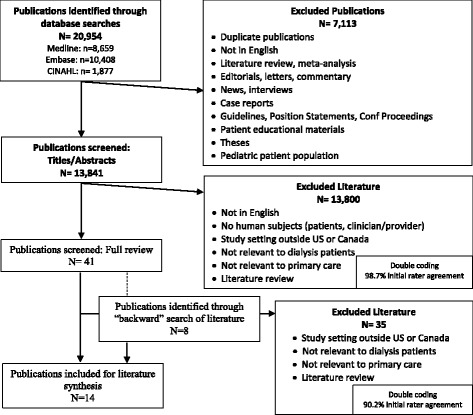
Identification of relevant papers included in literature synthesis

Keyword searches in electronic databases resulted in 13,841 citation hits, of which 10 met inclusion criteria. The large number of excluded citations and papers were largely due to the broad search terminology regarding primary care and prevention. Another 4 relevant articles were identified through backward search methods, resulting in a total of 14 articles regarding primary care services for dialysis patients (Fig. 2).

### Study designs of reviewed literature

Characteristics of the studies addressing primary care services for dialysis patients are described in Table 1. The overwhelming majority of articles were conducted in the US (*n* = 13, 93%) and one study was conducted in Canada. Of the 14 articles, there were 9 (64%) studies that conducted cross-sectional surveys or qualitative interviews with providers or patients, 4 retrospective cohort studies, and 2 quasi-experimental interventions (e.g., reporting results of quality improvement efforts). The cross-sectional surveys collected responses from a roughly equal share of nephrologists [14–17], non-nephrologist PCPs [15–17] and patients [17–20]. Four studies assessed dialysis patients’ receipt of primary care services using any combination of Medicare claims and registry sources (*n* = 3), or data obtained from medical chart review (*n* = 1) [21–24]. No studies assessed relationships between primary care services and clinical outcomes. Moreover, no clinical trials were identified addressing our key questions.

**Table 1:** Description of studies and measures on provision of primary care in dialysis patients

Author, Year	Research Question	Study Design	Setting/Location	Units of Observation	Outcome Categories
Wells, 1986 [16]	Practices of subspecialists and general internists in counseling about smoking and exercise	Physician survey	Internal Medicine Physicians, US, 1981	Physicians • nephrologists • primary care • trained generalists	• Time to deliver PC • Provision of various PC, by provider type
Nespor and Holley, 1992 [20]	Reliance on nephrology for health maintenance, management of non-renal chronic illness	Patient survey	Dialysis unit, US-Pittsburgh, PA	• Dialysis units, *n* = 1 • Dialysis patients, *n* = 74	• Has a PCP • Receipt of various PC, by provider type
Holley and Nespor, 1993 [19]	Reliance on and provision of general health care: in-center hemodialysis vs. peritoneal dialysis patients	Patient survey	Dialysis unit, US	• Dialysis units, *n* = 1 • Dialysis patients, *n* = 118	• Has/uses a PCP • Reliance of various PC, by provider type • Receipt of various PC, by provider type
Bender and Holley, 1996 [14]	Nephrologist PC practice patterns	Physician survey	Nephrologists, American Society of Nephrology, 1993	Practicing nephrologists, *n* = 233	• Patients have/use PCP • Provision of various PC • Time to deliver PC • Confidence in ability to provide PC
Alexander et al., 1998 [25]	Exploring experience obtaining medical care among dialysis patients	Patient interviews	Dialysis units in US-Northeast Ohio, Southeast Michigan, 1996–1997	• Dialysis units, *n* = 4 • Dialysis patients, *n* = 148	• Receipt of various PC, by provider type • Quality of overall medical care/satisfaction
Rodgers et al., 2000 [27]	Quality improvement effort to improve immunization rates	Quasi-experimental intervention	Dialysis units, in US-Nevada and Utah, 1998	• Dialysis units, *n* = 159 • Dialysis patients, *n* = 7004	• Receipt of vaccination
Winkelmayer, 2002 [24]	Use of preventive health care in patients before and after onset of dialysis	Retrospective cohort study (claims)	Medicare/Medicaid patients in US-New Jersey, 1990–1996	Dialysis patients, *n* = 1184	Receipt of PC preventive screening
Gilbertson et al., 2003 [21]	Effectiveness of influenza vaccination in dialysis patients	Retrospective cohort study (registry, claims)	Medicare, US, 1997–1999	Medicare dialysis patients, *n* = 125,500	Receipt of vaccination
Zimmerman, 2003 [17]	Opinions of nephrologists, family physicians and dialysis patients, concerning PC delivery to dialysis patients	Patient survey Provider surveys	Canada Physicians: national sample Patients: Toronto, Ottawa, Vancouver	• Nephrologists from Nephrology Society, *n* = 196 • Family Practitioners from random sample of urban practices, *n* = 167 • Patients from urban dialysis clinics, *n* = 147	• Time to deliver PC • Confidence in ability to provide PC • Opinions of who should provide PC • Written reports/updates to PCP • Provision of various PC, by provider type • Have/use PCP • Receipt of various PC, by provider type
Shah et al., 2005 [23]	Dialysis patient reliance on nephrologists for PC	Retrospective cohort study (Patient survey, chart review)	Dialysis unit, US, 1999–2001	• Dialysis units, *n* = 1 • Dialysis patients, *n* = 158	Has/use a PC
Claxton et al., 2010 [18]	Management of dialysis patient symptoms	Patient survey	Dialysis unit, 2007	• Dialysis units, *n* = 1 • Dialysis patients, *n* = 62	Receipt of various PC, by provider type
Duval, 2011 [26]	Effectiveness of intervention to increase vaccination rates	Quasi-experimental intervention	Dialysis units in US-Arkansas, Louisiana, Oklahoma, 2009–2010	• Dialysis units, *n* = 275 • Dialysis patients, *n* = 14,938	Receipt of vaccination (pre-intervention)
Green, 2012 [15]	Provider perceptions and practice patterns of symptom management and treatment	Provider survey	Dialysis units in Mid-Atlantic US; 2010	• Dialysis units, *n* = 9 • Nephrologists, *n* = 20 • Physician Assistants, *n* = 5 • Nurse Practitioners, *n* = 2	• Time to deliver PC • Opinions of who should provide PC • Provision of PC
McGrath et al., 2012 [22]	Effectiveness of influenza vaccine in dialysis patients	Retrospective cohort study (registry and claims)	Medicare, US, 1997–2001	Medicare dialysis patients, *n* = 464,317	Receipt of vaccination

*Abbreviations: PC* Primary care, *PCP* Primary care provider

Across studies included in this review, the dialysis patient was the most common unit of observation (*n* = 11 studies), followed by nephrologists (*n* = 4), and PCPs (*n* = 3). Interestingly, however, there was less variation among the types of outcomes assessed from the patient perspective, compared to the wider array of provider-oriented outcomes examined. Key measures about or reported by patients included whether patients had or used a PCP [14, 17, 19, 20, 23], the extent to which patients relied on primary care from nephrologists or non-nephrologists [19, 23] and the receipt of specific types of primary care services [17–22, 24–27]. In contrast, measures referencing or reported by providers assessed the estimated time devoted to and capacity for providing primary care to dialysis patients [14–17, 20], self-reported frequency of delivering specific types of primary care services [14–17, 19, 20, 25], nephrologists’ confidence in their ability to provide primary care [14, 17] and nephrologist and PCP opinions of who *should* provide primary care services [15, 17].

### Perceptions of provider roles and skills in primary care delivery

Four cross-sectional surveys examined patients and providers’ perceptions of PCP and nephrologists’ roles and capacity in the provision of primary care for dialysis patients (Table 2). Across these studies, most nephrologists’ were confident in their abilities serving as the primary care provider for dialysis patients [14, 17] or managing and treating symptoms, despite their perceived limited training and comfort in managing aspects of symptom management such as pain and depression [15].

**Table 2:** Patient and provider perceptions of primary care physician and nephrologist care

Author, Year	Perspective	Summary Finding
Bender & Holley, 1996 [14]	Nephrologists	Confident as primary care provider: 92%
Alexander et al., 1998 [25]	Dialysis patients	Physician type not associated with patient satisfaction of care
Zimmerman, 2003 [17]	Nephrologists	Confidence in abilities • Confident in own ability to provide primary care: 60% • Not very confident in Family Physician (FP) knowledge and training to provide primary care: 46% • Not very confident in FP available time to provide good primary care: 51%
		Roles and responsibilities • Nephrologist should not provide all PC for dialysis patients: 80% • Provision of primary care should be…equally split: 40% nephrologist has more responsibility than FP: 18% FP has more responsibility than nephrologist: 42%
	Family Practitioners	Confidence in abilities • Not very confident in Family Practitioner’s knowledge and training to provide PC: 40% • Not very confident in Family Practitioner’s available time to provide good PC: 62% • Nephrologist should not provide all PC for dialysis patients: 85%
		Roles and responsibilities • Nephrologist should not provide all primary care for dialysis patients: 85% • Provision of primary care should be…equally split: 34% nephrologist has more responsibility than FP: 17% FP has more responsibility than nephrologist: 40%
	Dialysis patients	Adequacy of their physicians training and time to address non-dialysis related problems • Training – Nephrologists: 46.5% Family physicians: 68.5% • Time – Nephrologists: 36.6% Family physicians: 68.5%
Green, 2012 [15]	Dialysis unit staff: • Nephrologists, • Physician Assistants • Nurse Practitioners	• Prior training on symptom treatment for pain (44%), depression (41%), sexual dysfunction (82%) • Non-nephrologist providers should be responsible for managing pain (59%), depression (82%), sexual dysfunction (63%) • Somewhat or very comfortable treating pain (69%), depression (69%), sexual dysfunction (48%)

*Abbreviations: PC* Primary care, *PCP* Primary care provider, *FP* Family Practitioner

In comparing nephrologists’ and family physicians’ perspectives, there was general agreement that primary care should not be exclusively provided by nephrologists [15, 17]. Yet, both nephrologists and family practitioners reported similar lack of confidence in family practitioners’ knowledge, training, and available time to provide quality primary care to dialysis patients [17]. Further, there was incongruence in provider perceptions related to trust, where nephrologists encouraged their patients to maintain relationships with their PCPs, but family practitioners were uncertain about nephrologists’ encouragement of PCP involvement [17].

From the patient perspective, overall satisfaction with medical care did not differ significantly by provider type [25]. In contrast to physicians’ perceptions, more patients believed family practitioners had the training (69%) and time (69%) to address their non-dialysis related symptoms compared to nephrologists (training: 46% and time: 37%) [17].

### Time, use, reliance and primary care service delivery

Physician estimates of delivery of primary care was determined via self-report, whereas receipt of primary care services was ascertained from patient self-report or administrative data collection (Table 3). Four studies assessed physician time dedicated to provision of primary care, all of which were reported by nephrologists. Most nephrologists reported spending at least a portion of their time delivering primary care, with varying reports of the amount of actual clinical time dedicated for primary care [14–17]. This variation may reflect secular trends. For example, only 8% of nephrologists in 1981 reported spending 75% or more of their time on general internal medicine [16] compared to nephrologists in 1993 who reported spending an average 38% of their practice time on primary care [14] and 85% nephrologists in 2010 who reported “moderate” to “a lot” of their time managing symptoms in dialysis patients [15].

**Table 3:** Provision and receipt of primary care services to chronic dialysis patients, by category^a^ and perspective

Perspective	Author, Year	Summary Findings
Time Delivering Primary Care		
Nephrologist (self-report)	Wells, 1986 [16]	• 8% spent >75% time on general internal medicine
	Bender & Holley, 1996 [14]	• 38% mean practice time on primary care issues
	Zimmerman, 2003 [17]	• 54% devoted >31% time to primary care
	Green, 2012 [15]	• 85% spend moderate - a lot time managing symptoms (general)
Have/Use/Reliance on Physician for Primary Care		
Nephrologist (self-report)	Wells, 1986 [16]	• 39% serve as PCP for ≥75% patients
	Bender & Holley, 1996 [14]	• 20% of patients have a PCP
PCP (self-report)	Zimmerman, 2003 [17]	Dialysis patients in PC practice: • 66% with no dialysis patients • 29% with 1–2 dialysis patients • 5% with 3–5 dialysis patients
Patient (self-report)	Nespor & Holley, 1992 [20]	• 20% have family doctor • 80% reliance on nephrologist for annual physical • 91% reliance on nephrologist for minor illness
	Holley & Nespor, 1993 [19]	• 29% of patients have family doctor • 59% of patients visited family practitioner in last 6 months • 81% reliance on nephrologist for annual physical, minor illness
	Zimmerman, 2003 [17]	• 87% have a family doctor, of which 65% visited family practitioner ≥2 times per year
	Shah, 2005 [23]	• General: 35% have PCP • 1-year before dialysis: 68% have PCP ﻿1-year after dialysis: 29% have PCP
Provision/Receipt of Primary Care Services: Patient Referrals^b^		
Nephrologist (self-report)	Bender & Holley, 1996 [14]	• Breast cancer screen - Mammography: 69% • Cervical cancer screen: 70% • Colon cancer screen: 43% • Endocrinologist: 25% • Cardiologist:76% • Gastroenterologist: 74%
	Zimmerman, 2003 [17]	• Breast cancer screen - Mammography: 30% • Cervical cancer screen: 28%
PCP (self-report)	Zimmerman, 2003 [17]	• Breast cancer screen - Mammography: 73% • Cervical cancer screen: 67%
Patient (self-report)	Nespor & Holley, 1992 [20]	By nephrologist: • Breast cancer screen - Mammography: 49% • Cardiologist:4% • Dermatologist: 9% • Gastroenterologist: 9% • Surgery (various): 36%
	Holley & Nespor, 1993 [19]	By nephrologist: • Breast cancer screen - Mammography: 40% • Cardiologist:36% • Endocrinologist: 27% • Gastroenterologist: 14%
Provision/Receipt of Primary Care Services: Counseling and Prevention^b^		
Nephrologist (self-report)	Bender & Holley, 1996 [14]	• Counseling: 79% • Breast exam: 52% • Colon cancer screen - Stool hemoccult: 73% • Offer immunization: 65%
	Zimmerman, 2003 [17]	• Counseling: 53% • Breast exam: 10% • Cervical cancer screen: 28% • Colon cancer screen - Stool haemoccult: 15% • Immunization: 74%
PCP (self-report)	Zimmerman, 2003 [17]	• Counseling: 77% • Breast exam: 78% • Cervical cancer screen: 67% • Stool haemoccult: 24% • Immunization: 88%
Patient (self-report)	Nespor & Holley, 1992 [20]	By nephrologist: Annual physical: 80%
		By non-nephrologist: • Eye exam: 58% • Gynecologic: 56% • Breast cancer screen - Mammography: 23%
	Holley & Nespor, 1993 [19]	By non-nephrologist: • Diabetic eye exam: 72% • Cervical cancer screen: 72% • Breast cancer screen - Mammography: 27%
	Zimmerman, 2003 [17]	Overall: • Breast cancer screen - Mammography:55% • Cervical cancer screen: 49%
		By nephrologist: • Annual physical: 21% By PCP: • Annual physical: 50%
Patient (claims, admin)	Rodgers, 2000 [27]	• Influenza vaccination from dialysis facility: 78% from neph office: 4% from non-neph office: 12% from other: 6%
	McGrath, 2012 [22]	• Influenza vaccination: 48%
	Winkelmayer, 2002 [24]	• Hemoglobin A1c testing: 11% • Diabetic eye exam: 76% • Breast cancer screen: 26% • Cervical cancer screen: 21% • Prostate cancer screen: 27%
	Gilbertson, 2003 [21]	• Influenza vaccination: 48%
	Duval, 2011 [26]	• Influenza vaccination: 77% • Pneumonia vaccination: 55%
Provision/Receipt of Primary Care Services: Acute Care, Disease and Symptom Management		
Nephrologist (self-report)	Bender & Holley, 1996 [14]	• General primary care: 90% • Treat acute minor illness: 85% • Disease management Hypercholesterolemia: 70% Diabetes: 90% Cardiac disease:75% Gastrointestinal Disease: 69%
	Zimmerman, 2003 [17]	• Treat minor illness: 72% • Prescribe meds – lipids: 82% – diabetes: 71% – heart: 74% – gastrointestinal: 59%
	Green, 2012 [15]	• Treating, “most” of the time Pain: 30% Depression: 19% Sexual dysfunction: 11%
PCP (self-report)	Zimmerman, 2003 [17]	• Treat minor illness: 91% • Prescribe meds – lipids: 78% – diabetes: 81% – heart: 82% – gastrointestinal: 85%
Patient (self-report)	Nespor & Holley, 1992 [20]	By nephrologist: • Minor illness: 91% • Diabetes: 63% • Heart disease:53% • Gastrointestinal disease: 88%
	Holley & Nespor, 1993 [19]	By nephrologist: • Diabetes: 73% • Heart disease:64% • Gastrointestinal disease: 86%
	Zimmerman, 2003 [17]	By PCP: • New problem (by PCP): 83% • Follow-up of ongoing problem (by PCP): 24% • Prescribed meds (by PCP): 51%
	Claxton, 2010 [18]	By nephrologist: • Physical symptoms: 13–70% • Mental health symptoms: 0% By PCP: • Physical symptoms: 20–63% • Mental health symptoms: 50–100%

*Abbreviation: PCP* non-nephrologist primary care provider

Notes:

^a^Reported findings may not be mutually exclusive and appear in multiple outcome categories

^b^For certain types of preventive care (e.g., cancer screening), physician referrals and direct delivery of preventive service are differentiated, appearing in separate outcome categories, where indicated

Dialysis patient use of PCPs was examined in several studies, from nephrologist, non-nephrologist provider, and patient perspectives [14, 16, 17, 19, 20, 23]. There was wide variation in patients either having or relying on non-nephrologist PCPs (ranges of 20–87% of patients) [14, 17, 19, 20, 23]. Nephrologists and patients reported generally similar extents (20–35%) of dialysis patients having a PCP [14, 19, 20, 23]. Patient-reported reliance on PCPs reflected these general trends, where 81% of patients relied on nephrologists for annual exams and minor illness [14] and reliance on PCPs diminished with dialysis vintage [19, 23].

General trends in provision and receipt of primary care differed by type of service. Overall, studies reported similar rates of primary care services delivered by nephrologist and PCPs. Within broad categories of primary care services, we found several discernible patterns. Nephrologists reported providing higher rates of consultative referrals [14] than those reported by patients [19, 20]. For counseling and preventive care services, nephrologists, PCPs, and patient reported generally consistent rates of preventive care (e.g., annual physicals, counseling, screening) delivered by both provider types [14, 17, 19, 20].

There were, however, some inconsistencies in reporting by data source. Patients reported lower rates of receipt of mammogram and cervical cancer screening than reportedly delivered by providers [17]. In contrast to the relatively high rates of patient- or provider-reported prevention services [14, 17, 19, 20], studies using administrative claims and medical chart data [21, 22, 24, 26, 27] found dialysis patients received suboptimal rates of preventive care. Rates of documented immunizations and vaccinations varied from 47% to 85% [21, 22, 26, 27]. In a cohort of dialysis patients enrolled in Medicare and Medicaid, Winkelmayer and colleagues (2002) found high rates of diabetic eye exams (76%) but low rates of hemoglobin A1c testing (11%) and cancer screening (21–27%).

Most of the studies examining physicians’ involvement in acute illness, symptom and disease management found similar rates of physician provision and patient receipt of care. For example, treatment of acute minor illness by nephrologists was reported by 72–85% of nephrologists and 91% of patients while 91% of PCPs also reported managing acute care illnesses for dialysis patients [14, 17, 20]. Similarly, high rates of nephrologist provision of disease management for diabetes, heart and gastrointestinal disease was found in patient- and nephrologist-reported surveys [14, 19, 20]; and nephrologist and PCP-driven medication management for hyperlipidemia, diabetes, heart disease, and gastrointestinal disease reported by these physician types [17]. Notable exceptions to these trends were found in patients reporting overwhelmingly high to exclusive use of PCPs for new problems and mental health symptoms, yet lower rates of PCP use for follow-up care [15, 17, 18].

## Discussion

In this review, we found a limited evidence base to inform our understanding of primary care services for chronic dialysis patients. From our systematic search of the healthcare literature, we found 14 studies spanning approximately three decades of research. Overall, we identified three themes related to primary care of dialysis patients: perceived roles of providers, estimated time in providing primary care, and the extent of dialysis patients’ use of primary care services (e.g., referrals, prevention, acute illness and disease management).

Among the findings in this literature, we found overall general agreement among providers that PCPs should be involved in primary care of dialysis patients. Despite this agreement, a potential barrier to realizing this may be the adequacy of time for PCPs to provide good primary care to dialysis patients and subsequent incongruent expectation and coordination between provider specialties. At the same time, the logistical barriers to accessing care and time burden of dialysis care for patients are a significant factor, which may also attribute to the majority of dialysis patients relying on primary care services from their nephrologists, despite their report of greater confidence in primary care delivered by non-nephrologist PCPs. These findings generally apply to all dialysis patients, as found in the few studies that explicitly assessed differences between in-center hemodialysis and home-based peritoneal dialysis patients. Although more PD than HD patients report having family practitioner [19], PD patients were less likely to see them over time [19] and have similarly low vaccination rates as HD patients [21]. With increasing use of home-based dialysis [8], future research should explore differences in patient care perceptions and utilization by treatment modality.

Across studies reported from a variety of patient and provider perspectives, we found varying degrees of consistencies of primary care service delivery and use with important implications on patient outcomes. On one hand, consistent reporting of delivery and receipt of primary care services may reflect accurate reporting across survey respondent types. On the other hand, high provision of care reported by nephrologists and PCPs may also suggest duplicative care. The pooled findings related to overall delivery and receipt of preventive care suggest potential gaps in care, where studies of administrative data found lower rates of preventive care services than found in studies relying on provider or patient self-report. The mechanisms underlying these inconsistent findings is unclear: this may be due to recall bias in self-reported data and potentially incomplete documentation of care in administrative data, but may also highlight discordant expectations and communication between patients, nephrologists, and PCPs that lead to gaps in care. The difficulty in communication between providers [17] is also exacerbated by a lack of streamlined electronic health records between health systems and dialysis units, resulting in a figurative chasm of information exchange. Further, the included studies were limited in their ability to assess the contribution of patient non-adherence to provider recommendations for preventive care, which may reflect patient behavior as a barrier to receipt of primary care services rather than inappropriate, duplicative, or missing care patterns by providers.

Although providers generally agreed that PCPs should be involved in primary care of dialysis patients, the optimal extent of this involvement is unclear. For example, many patients report having a non-nephrologist PCP, but simultaneously report reliance on nephrologists for referrals, counseling, preventive care, symptom and disease management; services that traditionally fall under the expertise of a PCP in the non-dialysis setting. There are no formal guidelines on the provision of primary care for individuals with ESRD, and the clinical benefit and cost-effectiveness of traditional health maintenance in this high-risk population remains controversial [28, 29]. While recent literature and consensus statements support reductions in cost-ineffective use of cancer screening in dialysis patients [30–32], the extent to which PCPs and nephrology providers are aware and/or agree with these recommendations is unclear.

## Conclusion

**Fig. 3:**
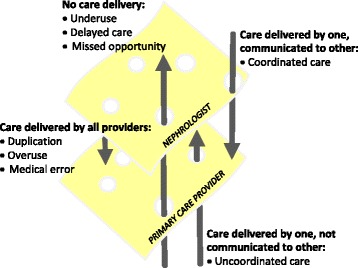
Typology of primary care fragmentation in the dialysis population

Altogether, these findings highlight opportunities for improvement and have important implications on the amount and quality of primary care that dialysis patients receive and on healthcare policy, as depicted in Fig. 3. This figure illustrates the Swiss Cheese Model of accident causation, used in engineering safety and healthcare and described here in the context of provider care and coordination of dialysis patients. Although dialysis patients likely see more providers than depicted here, our example presents a simple model of just PCPs and nephrologists. Each slice represents care delivered by a provider, where the holes reflect unprovided care. Patient care is completed neglected when holes between the slices of cheese align. For patient care, there is potential overlap in care that may be unnecessary and duplicative, placing time burdens on patients and their families and providers and financial burden on the healthcare system. In addition, the pooled, complementary findings suggest the possibility of a distinction in roles and responsibilities for some primary care services that result in non-duplicative, singular care. Importantly, this evidence base suggests the greater likelihood of gaps in care, leaving missed opportunities for clinical intervention and increasing the risk of adverse events, exacerbation of symptoms and disease, and increased healthcare utilization and costs.

While this is an emerging area of concern, it is useful to consider its importance and the findings from the extant literature in the context of changes in the healthcare delivery system for ESRD patients. Aided by improving survival rates, the US is experiencing growing ESRD prevalence and a shrinking nephrology and primary care workforce. Recent system-level interventions, such as Medicare’s managed care program demonstration for ESRD patients and the Comprehensive ESRD Care model aim to reorganize ESRD patient care through the collaboration of dialysis clinics, nephrologists and other US providers. This latter effort has resulted in Medicare-funded ESRD Seamless Care Organizations (ESCO) to improve care coordination and outcomes for Medicare beneficiaries. Thus, the research synthesized here foreshadows the challenges and opportunities for ESCOs to retool structures and processes of care across provider types to improve clinical and logistical complexity of care for patients on chronic dialysis.

Future research will want to examine the extent to which singular care is coordinated and communicated across patients, nephrologists and PCPs, as well as ways to optimize such multidisciplinary care for dialysis patients and improve outcomes. The extant literature presented here reveal gaps and opportunities for future research to augment and improve the evidence base. First, there is a paucity of claims-based research. The differential findings of studies based on administrative data versus individual self-reported observational studies, suggest that the truth is probably somewhere in between. Well-designed mixed methods research can examine the impacts of structures and processes of care coordination in a more nuanced way than quantitatively or qualitatively-based studies alone. Second, the studies examined in this review examined a limited set of outcomes in dialysis patients’ primary care. More studies linking the relationship between providers and patients’ use and receipt of primary care to outcomes (e.g., physical function, morbidity, hospitalization, mortality) will serve as important comparisons to the nascent ESCOs, whose value is yet to be determined.

## Abbreviations

CKD: Chronic kidney disease
ESCO: ESRD Seamless Care Organizations
ESRD: End-stage renal disease
MCC: Multiple chronic conditions
MeSH: Medical Subject Headings
PCP: Primary care physician

## References

[1] Diamantidis CJ, Powe NR, Jaar BG, Greer RC, Troll MU, Boulware LE (2011). Primary care-specialist collaboration in the care of patients with chronic kidney disease. Clin J Am Soc Nephrol.

[2] Greer RC, Ameling JM, Cavanaugh KL, Jaar BG, Grubbs V, Andrews CE, Ephraim P, Powe NR, Lewis J, Umeukeje E (2015). Specialist and primary care physicians’ views on barriers to adequate preparation of patients for renal replacement therapy: a qualitative study. BMC Nephrol.

[3] Abdel-Kader K, Greer RC, Boulware LE, Unruh ML (2014). Primary care physicians’ familiarity, beliefs, and perceived barriers to practice guidelines in non-diabetic CKD: a survey study. BMC Nephrol.

[4] Greer RC, Crews DC, Boulware LE (2012). Challenges perceived by primary care providers to educating patients about chronic kidney disease. J Ren Care.

[5] Linzer M, Myerburg RJ, Kutner JS, Wilcox CM, Oddone E, DeHoratius RJ, Naccarelli GV, Committee ASPW (2006). Exploring the generalist-subspecialist interface in internal medicine. Am J Med.

[6] Press MJ (2014). Instant replay--a quarterback’s view of care coordination. N Engl J Med.

[7] Berns JS, Szczech LA (2007). What is the nephrologist’s role as a primary care provider? We all have different answers. Clin J Am Soc Nephrol.

[8] US Renal Data System (2014). Annual data report: atlas of chronic kidney disease and end-stage renal disease in the United States.

[9] Parker MG, Ibrahim T, Shaffer R, Rosner MH, Molitoris BA (2011). The future nephrology workforce: will there be one?. Clin J Am Soc Nephrol.

[10] Salsberg E, Masselink L, Wu X (2014). The US nephrology workforce: developments and trends.

[11] National Quality Forum (2012). Multiple chronic conditions measurement framework.

[12] Retchin SM (2008). A conceptual framework for interprofessional and co-managed care. Acad Med.

[13] Institute of Medicine (1996). Primary care: America’s health in a new era.

[14] Bender FHH, J. L. (1996). Most nephrologists are primary care providers for chronic dialysis patients: results of a national survey. Am J Kidney Dis.

[15] Green JA, Mor MK, Shields AM, Sevik MA, Palevsky PM, Fine MJ, Arnold RM, Weisbord SD (2012). Renal provider perceptions and practice patterns regarding the management of pain, sexual dysfunction, and depression in hemodialysis patients. J Palliat Med.

[16] Wells KB, Lewis CE, Leake B, Schleiter MK, Brook RH (1986). The practices of general and subspecialty internists in counseling about smoking and exercise. Am J Public Health.

[17] Zimmerman DL, Selick A, Singh R, Mendelssohn DC (2003). Attitudes of Canadian nephrologists, family physicians and patients with kidney failure toward primary care delivery for chronic dialysis patients. Nephrol Dial Transplant.

[18] Claxton RN, Blackhall L, Weisbord SD, Holley JL (2010). Undertreatment of symptoms in patients on maintenance Hemodialysis. J Pain Symptom Manag.

[19] Holley JL, Nespor SL. Nephrologist-directed primary health care in chronic dialysis patients. Am J Kidney Dis. 1993;21(6):628–31. 10.1016/s0272-6386(12)80035-7 8503416

[20] Nespor SL, Holley JL. Patients on hemodialysis rely on nephrologists and dialysis units for maintenance health care. ASAIO J. 1992;38(3):M279–81. 10.1097/00002480-199207000-00037 1457865

[21] Gilbertson DT, Unruh M, McBean AM, Kausz AT, Snyder JJ, Collins AJ (2003). Influenza vaccine delivery and effectiveness in end-stage renal disease. Kidney Int.

[22] McGrath LJ, Kshirsagar AV, Cole SR, Wang L, Weber DJ, Sturmer T, Brookhart MA (2012). Influenza vaccine effectiveness in patients on hemodialysis: an analysis of a natural experiment. Arch Intern Med.

[23] Shah N, Dahl NV, Kapoian T, Sherman RA, Walker JA (2005). The nephrologist as a primary care provider for the hemodialysis patient. Int Urol Nephrol.

[24] Winkelmayer WC, Owen W, Glynn RJ, Levin R, Avorn J (2002). Preventive health care measures before and after start of renal replacement therapy. J Gen Intern Med.

[25] Alexander GC, Sehgal AR (1998). Dialysis patient ratings of the quality of medical care. Am J Kidney Dis.

[26] Duval L, George C, Hedrick N, Woodruff S, Kleinpeter MA (2011). Network 13 partnership to improve the influenza, pneumococcal pneumonia, and hepatitis B vaccination rates among dialysis patients. Adv Perit Dial.

[27] Rodgers DJ, Karp SK, Woodruff SD, Wright LD, Stiles SK (2000). Influenza immunization rates in the intermountain end-stage renal disease network (network 15). Adv Ren Replace Ther.

[28] Holley JL (2011). Do dialysis patients need screening colonoscopies and mammograms?. Semin Dial.

[29] LeBrun CJ, Diehl LF, Abbott KC, Welch PG, Yuan CM (2000). Life expectancy benefits of cancer screening in the end-stage renal disease population. Am J Kidney Dis.

[30] American Board of Internal Medicine (ABIM). American Society of Nephrology: Don’t perform routine cancer screening for dialysis patients with limited life expectancies without signs or symptoms (Released April 4, 2012). In Choosing Wisely. 2016. http://www.choosingwisely.org/clinician-lists/american-societynephrology-routine-cancer-screening-for-dialysis-patients. Accessed 16 July 2017.

[31] Chertow GM, Paltiel AD, Owen WF, Lazarus JM (1996). Cost-effectiveness of cancer screening in end-stage renal disease. Arch Intern Med.

[32] Wong G, Howard K, Chapman JR, Craig JC (2008). Cost-effectiveness of breast cancer screening in women on dialysis. Am J Kidney Dis.

